# Optimal Timing of Cholecystectomy for Acute Cholecystitis: A Retrospective Cohort Study

**DOI:** 10.7759/cureus.28548

**Published:** 2022-08-29

**Authors:** Shelbie D Kirkendoll, Edward Kelly, Kristina Kramer, Reginald Alouidor, Eleanor Winston, Tyler Putnam, Gabriel Ryb, Nicolas Jabbour, Aixa Perez Coulter, Tovy Kamine

**Affiliations:** 1 Department of General Surgery, Valley Health System, Las Vegas, USA; 2 Acute Care Surgery, University of Massachusetts Medical School Baystate, Springfield, USA

**Keywords:** post operative complications, bile leak, length of stay, biliary diseases, cholecystitis, laporoscopic cholecystectomy, early laparoscopic cholecystectomy, cost, acute calculus cholecystitis, gallbladder

## Abstract

Background

Laparoscopic cholecystectomy performed less than 72 hours from hospital admission for acute cholecystitis has shown to decrease hospital cost without an increase in length of stay (LOS). Very few studies have examined clinical and cost outcomes of performing cholecystectomy less than 24 hours from hospital admission. The aim of this study was to examine the cost and LOS of laparoscopic cholecystectomy performed on an early (less than 24 hours from admission) and late (more than 24 hours from hospital admission) basis.

Methods

We performed a retrospective observational study of 569 patients at Baystate Medical Center, Springfield, USA, who underwent urgent laparoscopic cholecystectomy for acute cholecystitis between January 1, 2018 and February 28, 2020. We evaluated preoperative/postoperative LOS, operative duration, hospital cost, and patient complications.

Results

468 patients underwent urgent laparoscopic cholecystectomy for acute cholecystitis during our study period. Early cholecystectomy (less than 24 hours from admission) had an overall decreased LOS (43.6 hours versus 102.9 hours, p-value < 0.01) and decreased hospital cost ($23,736.70 versus $30,176.40, p-value < 0.01) compared to late cholecystectomy (more than 24 hours from admission). There was also a significantly higher rate of bile leak in patients who underwent surgery more than 24 hours from hospital admission compared to those who had surgery less than 24 hours from admission (5.9% versus 0.4%, p-value < 0.01). Additionally, those procedures performed greater than 24 hours from hospital admission were significantly more likely to be converted to an open procedure (6.9% versus 2.2%, p-value = 0.02).

Conclusion

Urgent laparoscopic cholecystectomy performed within 24 hours of hospital admission for acute cholecystitis decreased hospital cost, LOS, and operative complications in our institution's patient population. Our data suggests that performing laparoscopic cholecystectomy within 24 hours of hospital admission would be beneficial from a patient and hospital standpoint.

## Introduction

Prior to the advent of laparoscopic surgery in the 1980s, it was standard to perform cholecystectomy for acute cholecystitis less than seven days from hospital admission [1-2]. Today, 90% of cholecystectomies in USA are performed laparoscopically [3]. However, in the laparoscopic era, the timing of cholecystectomy has been heavily debated. A consensus eventually developed to perform cholecystectomy for acute cholecystitis early, or less than 72 hours from admission, and to perform cholecystectomy for symptomatic cholelithiasis in an interval fashion, or six-to-12 weeks from initial presentation [2].

In USA, cholecystectomy is the most common procedure done by general surgeons each year [4]. As many hospitals in USA function as accountable care organizations (ACOs), staying aware of hospital costs is important since hospitals in an ACO model are paid for quality of care and not quantity [5]. In addition, as Medicare ties reimbursement to patient satisfaction [6], it is critical for hospital systems to focus on providing the best care for the patient, both in terms of cost and patient experience.

We set out to perform a retrospective cohort study investigating the difference in length of stay (LOS) and hospital cost in patients undergoing laparoscopic urgent cholecystectomy. A 24-hour cut-off was chosen based on previous data showing that 24 hours was equivalent to 72 hours from a complication and patient care perspective [7]. In particular, we sought to determine if cholecystectomy performed less than 24 hours from hospital presentation for patients who present to the emergency room with acute cholecystitis is beneficial from a financial perspective.

This article was previously presented as an oral presentation at the 68th annual meeting of the Massachusetts chapter of the American College of Surgeons on December 4, 2021 and as a poster presentation at the 2021 AMA Research Challenge from October 21 to 23, 2021.It was presented to the Baystate Medical Center IRB under the name "Process of Care for Urgent Cholecystectomies: A Quality Improvement Study" and assigned the number 1655533-2.

## Materials and methods

Data collection

We performed a retrospective chart review on patients who underwent urgent laparoscopic cholecystectomy for acute cholecystitis between January 1, 2018 and February 28, 2020 at Baystate Medical Center, Springfield, USA. Patients were excluded if they were diagnosed with gallstone pancreatitis or choledocholithiasis, underwent pre-operative endoscopic retrograde cholangiopancreatography (ERCP), carried a diagnosis of ischemic heart disease, or were on non-reversible therapeutic anticoagulation. These were all noted to be unavoidable reasons for delaying cholecystectomy less than 24 hours from admission. In total, 158 patients were excluded to ensure that the LOS and financial analyses were between like groups. Our final analysis included 569 patients.

The hospital stays for all patients were divided into three phases (preoperative, operative, and postoperative LOS). Preoperative LOS was defined as the time from admission through the emergency department until operative start time. Operative LOS was defined as operative start time to operative end time. Postoperative LOS was defined as operative end time to time of discharge from the hospital. Total LOS was defined as the time of admission to the time of discharge. Post-operative complications included mortality, deep vein thrombosis, pulmonary embolism, surgical site infection, bile leak, and conversion to an open procedure. Cost and payment data were gathered from Baystate Medical Center McKesson billing system. Of note, individual payers and payer mix were not able to be identified from the data. For the purposes of cost calculations, 66 patients without cost data were also excluded.

Data analysis

We evaluated differences in patient characteristics for urgent cholecystectomy patients across groups (less than 24 hours versus more than 24 hours preoperative LOS). Descriptive statistics were calculated, including percentages for binary categorical variables and means and standard deviations for continuous variables. T-test was used for continuous variables and Fisher exact test for categorical variables. Variables collected included demographics (i.e. age, sex) as well as clinical characteristics (i.e. comorbidities, time of day). Outcomes of interest included LOS and cost data. Statistical significance was defined as an alpha of 0.05. Data was analyzed using STATA 16 (StataCorp, College Station, USA).

Data are available on reasonable request. The data are stored as de-identified participant data which are available on request to Aixa Perez Coulter (). 

## Results

Demographics

Table 1 shows the demographics of the 411 patients in this study. We found there was no significant difference in age, sex, or prevalence of diabetes in patients undergoing early versus late cholecystectomy. 

**Table 1:** Patient demographics of early (less than 24 hours) versus late (more than 24 hours) cholecystectomy n: Number of patients; SD: Standard deviation; LOS: Length of stay

	Early cholecystectomy (<24 hours preoperative LOS)	Late cholecystectomy (>24 hours preoperative LOS)	p-value (early vs late)
Number of patients (n)	225	186	
Age, mean (SD)	46.7 (17.3)	48.6 (17.7)	0.27
Sex, n (%)			
Female, n (%)	161 (71.6)	128 (68.8)	
Male, n (%)	64 (28.4)	58 (31.2)	0.55
Diabetes, n (%)	26 (11.6)	28 (15.1)	0.30

Table 2 shows the clinical characteristics and cost information of patients undergoing early versus late cholecystectomy.

**Table 2:** Clinical characteristics and cost data of early (less than 24 hours) versus late (more than 24 hours) cholecystectomy n: Number of patients; SD: Standard deviation; LOS: Length of stay; OR: Operating room

	Early cholecystectomy (<24 hours preoperative LOS)	Late cholecystectomy (>24 hours preoperative LOS)	p- value (early vs late)
Number of patients (n)	225	186	
Weekend, n (%)	51 (22.7)	51 (27.5)	0.77
Timing of operation			
Time of day, n (%)			
-0700-1500, n (%)	61 (27.1)	59 (31.7)	
-1500-2300, n (%)	87 (38.7)	64 (34.4)	
-2300- 0700, n (%)	77 (34.2)	63 (33.9)	0.54
Total LOS (hours), mean (SD)	43.6 (36.9)	102.9 (95.5)	<0.01
-Preoperative LOS (hours), mean (SD)	12.6 (9.9)	52.5 (35.4)	<0.01
-Postoperative LOS (hours), mean (SD)	29.3 (36.0)	41.9 (59.9)	0.01
Operative duration (hours), mean (SD)	1.7 (1.8)	1.2 (3.5)	0.07
Cost information			
Cost, mean (SD)	$23,736.70 ($7,460.70)	$30,176.40 ($22,546.80)	<0.01

Timing of cholecystectomy

Among patients who underwent urgent cholecystectomy, there were no significant differences in day of the week or time of day of the operation between those undergoing early (less than 24 hours from admission) versus late (more than 24 hours from admission) cholecystectomy. 

LOS

Patients who underwent early cholecystectomy (less than 24 hours) had significantly shorter overall mean LOS compared to those who underwent late cholecystectomy (43.6 hours versus 102.9 hours, p < 0.01). In addition to their decreased mean preoperative LOS (12.6 hours versus 52.5 hours, p < 0.01), the patients in the early cholecystectomy group had a significantly shorter mean postoperative LOS as well (29.3 hours versus 41.9 hours, p = 0.01). There was no significant difference in operative duration time between the two groups (1.7 hours versus 1.2 hours, p = 0.07).

Cost data

Patients who underwent early cholecystectomy had significantly decreased mean total hospital cost compared to late cholecystectomy patients ($23,736.70 versus $30,176.40, p < 0.01).

Patient complications

Table 3 shows patient complications of early (less than 24 hours) versus late (more than 24 hours) cholecystectomy. There was a significantly higher rate of bile leak in patients who underwent surgery >24 hours from hospital admission compared to those who had surgery less than 24 hours from admission (5.9% versus 0.4%, p < 0.01). Additionally, those procedures performed more than 24 hours from hospital admission were significantly more likely to be converted to an open procedure (6.9% versus 2.2%, p = 0.02). There was no significant difference in the rate of DVT (deep vein thrombosis), PE (pulmonary embolism), surgical site infection, or mortality between LOS less than 24 hours and LOS more than 24 hours. Of note, the mortality that occurred during this study was unrelated to the procedure.

**Table 3:** Patient complications of early (less than 24 hours) versus late (more than 24 hours) cholecystectomy n: Number of patients; DVT: Deep vein thrombosis; PE: Pulmonary embolism

	Early cholecystectomy (<24 hours preoperative LOS)	Late cholecystectomy (>24 hours preoperative LOS)	p-value (early vs late)
Number of patients (n)	225	186	
DVT/PE, n (%)	0 (0)	2 (1.1)	0.12
Surgical site infection, n (%)	0 (0)	1 (0.5)	0.27
Bile leak, n (%)	1 (0.4)	11 (5.9)	<0.01
Conversion to open, n (%)	5 (2.22)	13 (6.99)	0.019
Death, n (%)	0 (0)	1 (0.54)	0.271

## Discussion

Previous studies have shown that early surgical intervention for gallbladder pathology (less than 72 hours from hospital admission) decreases hospital costs without an increase in operative duration or surgical complications [8-10]. Additionally, early operative intervention has been shown to decrease operative mortality, duration of antibiotic therapy, and cost of hospital stay [9,11]. This contrasts with cholecystectomy performed more than 72 hours from presentation, which has been suggested by one study to be a more difficult procedure and actually increases the chance of emergent operative intervention due to failed conservative management [1]. Further, for patients with cholecystitis, early cholecystectomy (less than 24 hours) has been shown to decrease the length of total hospital stay and operative duration outside of the US healthcare system [7,12].

In our patient population, 54% of cholecystectomies were done within 24 hours of hospital presentation. The other 46% of patients underwent cholecystectomy more than 24 hours after presentation and did not fit the aforementioned reasons for delaying cholecystectomy (i.e., gallstone pancreatitis, choledocholithiasis, heart disease, or those on non-reversible therapeutic anticoagulation). These patients with predetermined reasons for late cholecystectomy were excluded from our analysis to ensure a similar group comparison for LOS and financial analyses. For patients without an obvious reason for late cholecystectomy, we found that performing laparoscopic cholecystectomy within 24 hours of hospital admission decreased the risk of bile leak and conversion to an open procedure. This is likely because a longer preoperative stay increased inflammation in these patients with cholecystitis and made the operation more technically difficult.

We found that the cost of performing laparoscopic cholecystectomy within 24 hours is significantly lower than the cost of late cholecystectomy. Early cholecystectomy for cholecystitis significantly decreased the LOS both preoperatively and postoperatively compared to late cholecystectomy. This suggests that hospitals would benefit from both capacity management and financial standpoints to perform cholecystectomy for acute cholecystitis within 24 hours of presentation to the emergency department. The cost savings from performing all cholecystectomies within 24 hours in our study would be $1,197,784.20 over the course of the 25 months of the study or $598,892.10 per year. This suggests that hiring additional surgical coverage may be cost-effective if it enables a significantly larger proportion of these cases to be performed within 24 hours. Further, previous studies have shown that decreased hospital LOS may contribute positively to patient satisfaction, which is likely another positive result of early cholecystectomy [13].

There are a number of limitations of our study. One such limitation is that these results may be specific to just our institution and may not be generalized to every institution in USA, as our study was conducted at a single institution and included only 569 patients. In our cost analysis, we recognize that the hospital costs are unique to our institution and thus our results may not be generalized. Further, we did not investigate payer mix and actual payments or hospital charges which may change the cost-effectiveness cut-off for urgent cholecystectomy at any individual institution. Further study is necessary to determine the effect of the payer mix on hospital financials for this very common operation. In addition, the decision to delay cholecystectomy more than 24 hours from hospital admission is complex. It includes multiple variables such as external factors (personnel, operating room availability, etc.) in addition to patient specific factors (comorbidities, overall clinical picture, etc.). Though we excluded patients with obvious reasons for a delay, there may be additional meaningful factors such as the availability of an operating room or surgical staff and the protocols for booking non-elective operations that we did not include in our study, which could explain differences between the two groups.

## Conclusions

Current literature has consistently shown that cholecystectomy for acute cholecystitis should be performed within 72 hours of hospital admission to decrease operative complications and mortality, LOS, and hospital costs. This benefit has also been demonstrated at the 24 hour mark as well. However, there is still no evidence favoring early laparoscopic cholecystectomy (less than 24 hours) in the US healthcare system.

We set out to determine if laparoscopic cholecystectomy for acute cholecystitis should be performed within 24 hours from hospital admission from a cost and LOS perspective. Overall, we found that in our single-center study, urgent cholecystectomy performed within 24 hours of hospital admission decreased LOS and hospital cost without increasing operative duration. We additionally found that early cholecystectomy (less than 24 hours) had a significantly lower operative risk, including risk of bile duct injury and conversion to an open procedure. This data together suggests that for both patient and hospital, the ideal course of treatment for acute cholecystitis is to perform cholecystectomy within 24 hours of presentation.
